# Age-related deficit in a bimanual joint position matching task is amplitude dependent

**DOI:** 10.3389/fnagi.2015.00162

**Published:** 2015-08-21

**Authors:** Matthieu P. Boisgontier, Stephan P. Swinnen

**Affiliations:** ^1^Movement Control and Neuroplasticity Research Group, Department of Kinesiology, Biomedical Sciences Group, KU LeuvenLeuven, Belgium; ^2^Leuven Research Institute for Neuroscience and Disease (LIND), KU LeuvenLeuven, Belgium

**Keywords:** aging, joint position sense, proprioception, humans, bimanual matching task

## Abstract

The cognitive load associated with joint position sense increases with age but does not necessarily result in impaired performance in a joint position matching task. It is still unclear which factors interact with age to predict matching performance. To test whether movement amplitude and direction are part of such predictors, young and older adults performed a bimanual wrist joint position matching task. Results revealed an age-related deficit when the target limb was positioned far from (25°) the neutral position, but not when close to (15°, 5°) the neutral joint position, irrespective of the direction. These results suggest that the difficulty associated with the comparison of two musculoskeletal states increases towards extreme joint amplitude and that older adults are more vulnerable to this increased difficulty.

## Introduction

Perception is an interpretation of physical reality. Proprioception is the perception of our body state in the absence of vision (Goble et al., 2009; Proske and Gandevia, 2012; Boisgontier and Swinnen, 2014). This state is defined by position, movement, and muscle force or tension. Interpretation of this state is based on the processing of information from peripheral receptors and motor efference copies (Proske and Gandevia, 2012). The proprioception that interprets body segment position is called joint position sense.

Joint position sense has been widely investigated in the context of aging (for a review, see Goble et al., 2009). Some of these investigations used dual-task paradigms to reveal that the cognitive load associated with joint position sense increased with age (Boisgontier et al., 2012; Goble et al., 2012b). However, such increased load does not necessarily result in an impaired performance in a joint position matching task, which is the typical task used to test joint position sense. Indeed, a number of studies reported the absence of an age effect (Jordan, 1978; Stelmach and Sirica, 1986; Batavia et al., 1999; Deshpande et al., 2003; Pickard et al., 2003; Goble et al., 2012a; Wang et al., 2012; Boisgontier and Nougier, 2013a; Schmidt et al., 2013). The factors that determine whether age will impact performance on a matching task are still unclear. These factors can be associated with the individual or with the context and features of the task. The level of physical activity has been shown to be one element in these predictors (Ribeiro and Oliveira, 2007; Adamo et al., 2009), and joint amplitude may be another.

In young adults, matching errors increase with target amplitude (Allen and Proske, 2006; Goble et al., 2006; Goble and Brown, 2008; Rincon-Gonzalez et al., 2011) but are not dependent on target direction (Walsh et al., 2013). The effect of amplitude on position errors could be surprising as joint proprioceptor response to passive movement increases towards the range of motion limits (Burke et al., 1988). However, paradigms of these studies allowed the use of an internal representation of the movement of the target limb (including duration and speed) to perform the matching task (Allen and Proske, 2006; Goble et al., 2006; Goble and Brown, 2008; Rincon-Gonzalez et al., 2011). Therefore, the effect of amplitude on joint position sense reported in these studies could also result from an effect on movement sense and if speed was kept constant across trials, from a tradeoff between accuracy and speed (Fitts, 1954; Fitts and Peterson, 1964).

Here, we propose that the effect of amplitude could be explained by examining the difficulty to match the perceptions of two musculoskeletal states that are different in nature (passive vs. active). To match position of one hand with the other hand in the absence of vision, we rely on proprioceptive signals generated from both limbs (Izumizaki et al., 2010). As described by Walsh et al. (2013), the brain is likely to compare proprioceptive afferent signals from the two limbs and when the difference between the signals is at a minimum, the positions are assumed to match each other. Furthermore, recent studies showed that the brain prioritizes the processing of information from both limbs over information from a single limb, resulting in better performance (Boisgontier and Nougier, 2013b; Savage et al., 2015). Therefore, when proprioceptive information associated to muscle contraction is present only in one limb, this information may not be considered as relevant information to the matching process. In other words, in this case, contraction-related information can be considered as noise. Since the intensity of the contraction increases towards range of motion limits to counter the resistance of passive tissues, contraction-related noise increases concurrently. In other words, amplitude would impact difficulty, i.e., the cognitive load of the task, and may trigger performance decline in older adults as they consistently function at a higher level of processing than young adults (Ward and Frackowiak, 2003; Heuninckx et al., 2008; Goble et al., 2010, 2012b; Boisgontier et al., 2012) and as their cognitive reserve is more limited than in young adults (Boisgontier et al., 2013).

To test whether amplitude and/or direction affect the ability to match two musculoskeletal states, young and older adults performed a bimanual joint position matching task with three amplitudes and two directions. Based on the aforementioned evidence, we hypothesized that the effect of age is dependent on amplitude but not direction.

## Materials and Methods

### Participants

Thirty young [21.1 ± 1.5 (19–24) years, mean ± SD (range); 14 females] and 28 older [69.4 ± 5.3 (61–82) years; 15 females] healthy volunteers participated in the study. All participants were right-handed according to the Edinburgh Handedness Inventory (Oldfield, 1971). The average lateralization quotient was similar between young and older adults (+91 ± 15 vs. +90 ± 19, respectively, with a +100 score representing extreme right-hand preference and a −100 score representing extreme left-hand preference). All participants had normal or corrected-to-normal vision, and none reported neurological, psychiatric, cardiovascular, or neuromuscular disorders. Older participants were screened for cognitive impairments with the Montreal Cognitive Assessment test using the standard cutoff score of 26 (Nasreddine et al., 2005). All participants gave their written informed consent, and procedures were performed according to guidelines established by the ethics committee for biomedical research at the KU Leuven, and in accordance with the WMA International Code of Medical Ethics (World Medical Association Inc., 1964).

### Apparatus

The apparatus used to test wrist joint position sense consisted of two separate, adjustable units (left and right), both equipped with a forearm support and a manipulandum for the palm (Boisgontier et al., 2014). Motion of the right wrist joint (passive limb) was induced by an AC Servo Motor (AMK DV764, Goedhard PMC, Helmond, Netherlands) mounted underneath the right hand unit and coupled to the rotating shaft of the manipulandum via a 1:10 reducer (Alpha LP120 Gearbox). The left hand piece was constructed similarly but allowed free flexion-extension wrist movement (active limb). Shaft encoders (accuracy = 0.088°) were connected to the rotating axis to record angular displacement of the left wrist and the right wrist. Data were sampled at 1000 Hz (Signal software 4.0, Cambridge Electronic Design, Cambridge, UK) and low-pass filtered (second-order Butterworth, cut-off frequency 8 Hz, zero-lag). The angular displacement signals of the two hand pieces were stored for offline analysis.

### Procedures

To control for muscle history effects (i.e., thixotropy; Proske et al., 1993), wrist muscle flexors and extensors were conditioned by asking participants to perform isometric contractions for 2 s at approximately half-maximal intensity at the start of the experiment (Allen et al., 2010). To perform the matching task, participants were seated in front of the apparatus with their shoulders in slight abduction (20°), elbows at 90°, forearms supported in neutral prosupination, and wrists in a neutral flexion-extension position. Vision was occluded by opaque goggles. They were instructed to match the right-hand position (target limb) with their left hand (matching limb) as accurately as possible at a self-selected movement speed, with the possibility of final submovements. The task was completed when participants stopped moving the matching limb for more than 1 s. This final position of the limbs was used to compute the dependent variable. The matching task was performed with three amplitudes (5, 15, and 25°) and two directions (flexion and extension). The target limb was positioned through an indirect movement including various flexions and extensions ranging from −25 to 25° to prevent movement-based matching. Each condition was performed three times. Experimental trials were administered in random order.

Electromyographic (EMG) activity from the right flexor carpi radialis and extensor carpi radialis muscles of the wrist was monitored throughout the experiment to control for the absence of muscle activity. EMG signals were amplified (×1000), filtered (4–500 Hz), and sampled at 1000 Hz. When muscle activity was observed in the EMG before the beginning of a trial, participants were instructed to relax their wrist.

### Data Analysis

Performance in the matching task was assessed using amplitude total error. The total error, also called total variability, root mean square error, or simply *E*, is explained equally by the response variability and bias (total error^2^ = variable error^2^ + constant error^2^; Henry, 1975). The total error was therefore preferred over the absolute error, a more complex relationship between the response variability and bias that complicates the determination of the relative contribution of each component (Schutz and Roy, 1973). Total error was defined according to the following formula:

$$\sqrt{\frac{1}{n}\times \sum {\left(x_{i}-t\right)}^{2}}$$

where *x*_i_ is the score on trial i, *t* is the target (*t* = −25, −15, −5, 5, 15, or 25°) and *n* is the number of trials (*n* = 3).

### Statistical Analyses

To test whether amplitude or direction impacted the effect of aging on joint position sense, total errors were analyzed by a 2 × 3 × 2 analysis of variance (ANOVA) with the factors Age (Young adults, Older adults), Amplitude (5, 15, 25°), and Direction (Flexion, Extension). Level of significance (α) was set at *p* = 0.05. When the ANOVA revealed significant effects, *post hoc* tests (Tukey HSD, which corrects for multiple comparisons) were conducted to identify the loci of these effects. Partial eta squared values (${\text{η}}_{p}^{2}$) were reported to indicate small (≥0.01), medium (≥0.06), and large (≥0.14) effect sizes (Sink and Stroh, 2006).

## Results

**Figure 1:**
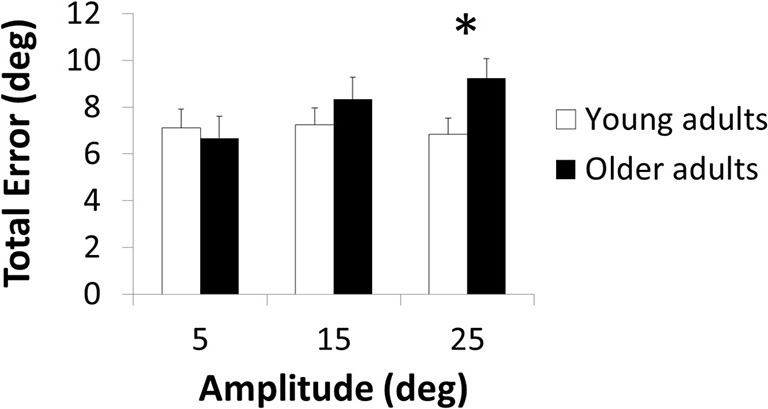
**Total error as a function of the amplitude of the target position in young and older adults.** *indicates significant difference.

The three-way ANOVA demonstrated a significant main effect of Amplitude [*F*_(2,112)_ = 5.06; *p* = 0.008; ${\text{η}}_{p}^{2}$ = 0.08] with greater total error in the 25-degrees than 5-degrees condition (*p* = 0.011). Main effects of Age [*F*_(1,56)_ = 2.75; *p* = 0.103; ${\text{η}}_{p}^{2}$ = 0.05] and Direction [*F*_(1,56)_ = 0.90; *p* = 0.348; ${\text{η}}_{p}^{2}$ = 0.02] were not significant. The Age × Amplitude interaction was significant [*F*_(2,112)_ = 5.39; *p* = 0.006; ${\text{η}}_{p}^{2}$ = 0.09; Figure 1] but not the Age × Direction [*F*_(1,56)_ = 0.28; *p* = 0.600; ${\text{η}}_{p}^{2}$ < 0.01] and three-way interaction [*F*_(2,112)_ = 0.37; *p* = 0.693; ${\text{η}}_{p}^{2}$ < 0.01]. *Post hoc* analyses revealed an age-related total error increase in the 25-degrees condition (*p* = 0.049) but not in the other amplitude conditions (*p* > 0.692; Figure 1).

## Discussion

Here, we investigated whether the effect of age in a bimanual wrist joint position matching task was dependent on target amplitude and direction. Results revealed an age-related deficit when the target limb was positioned far (25°, *p* = 0.049) from but not close to (15°, *p* = 0.692; 5°, *p* > 0.999) the neutral position, irrespective of direction (*p* = 0.693).

### Sensitivity to an Increase in Task Difficulty in Young and Older Adults

The age-related decline in matching performance observed in the highest amplitude suggested that the cognitive load resulting from the processing of proprioceptive input reached a threshold in this condition, and triggered a decline in matching performance. We believe that such a decline is explained by a combination of the points reported below. Older adults consistently function at a higher level of processing than young adults in order to compensate for a decreased signal-to-noise ratio (Ward and Frackowiak, 2003; Heuninckx et al., 2008; Goble et al., 2010, 2012b; Boisgontier et al., 2012). Furthermore, their cognitive reserve is more limited than in young adults (Boisgontier et al., 2013). Therefore, older adults may be more sensitive to an increase in task difficulty. Such an increase in difficulty can result from the addition of information that is not relevant to the task such as noise at the peripheral and processing levels.

### Increased Noise at the Proprioceptor Level

The decrease in signal-to-noise ratio may originate at the proprioceptor level. Due to age-related proprioceptors alteration (Bolognia, 1995; Liu et al., 2005; Aydoğ et al., 2006), the firing rate may show greater variance in older adults, thereby replicating observations made for motor neurons (Laidlaw et al., 2000). Such an age-related increase in the variance of the firing rate would decrease the signal-to-noise ratio. In addition, the standard deviation of motor-neuronal firing has been shown to increase with its mean level (Clamann, 1969; Matthews, 1996). Assuming that standard deviation perception-neuronal firing also increase with its mean level, and as the joint proprioceptor firing rate increases towards range of motion limits (Burke et al., 1988), the age-related decrease in the signal-to-noise ratio would be amplified towards range of motion limits.

### Increased Noise at the Processing Level

At the processing level, the hypothesis stating that our findings result from a speed-accuracy tradeoff does not hold, as final submovements were allowed. Additionally, the random flexion-extension movements performed during positioning of the target limb prevented the participant from matching movement features, and only allowed matching of the position *per se*. We propose that this decline in performance was instead accounted for by an amplitude-dependent amplification of the difficulty to match the perceptions of two musculoskeletal states that are different in nature (passive vs. active). The brain prioritizes the processing of information from both limbs over information from a single limb (Boisgontier and Nougier, 2013b; Savage et al., 2015). In our bimanual matching task, proprioceptive information associated to muscle contraction is only generated in one limb (active matching limb) and may therefore be considered as noise. Since the intensity of the contraction increases towards range of motion limits, matching performance would decline as the target limb moves away from the neutral position, which supports our findings.

Additionally, although not assessed here, range of motion has been shown to be limited in older adults (Chaparro et al., 2000). Therefore, the intensity of the contractions may be higher in older than in young adults, especially when the target limb was positioned far from the neutral position. This increased intensity of the contractions would amplify the noise at both the peripheral and processing level and explain the age-related deficit observed in our study.

### No Age-Related Deficit in the Lower Amplitudes

The absence of a difference in matching performance between young and older adult in the lower amplitudes supports studies demonstrating the absence of age-related deficits in joint position sense (Jordan, 1978; Stelmach and Sirica, 1986; Batavia et al., 1999; Deshpande et al., 2003; Pickard et al., 2003; Goble et al., 2012a; Wang et al., 2012; Boisgontier and Nougier, 2013a; Schmidt et al., 2013). These results demonstrate that older adults are able to sense position to the same degree as young adults under certain circumstances. Specifically, it shows that the accuracy of proprioceptor information is either robust against aging or altered by aging but compensated for by central mechanisms. The latter is more likely as age-related changes in muscle (Liu et al., 2005), joint (Aydoğ et al., 2006) and skin (Bolognia, 1995) receptors are thought to reduce the signal-to-noise ratio by decreasing quantity and/or intensity of their output and increasing sensory noise (Speers et al., 2002). Moreover, the greater recruitment of neural resources in older relative to young adults observed in motor tasks supports the idea of a compensation mechanism (Ward and Frackowiak, 2003; Heuninckx et al., 2008; Goble et al., 2010).

## Author Contributions

Experimental conception and design: MPB. Experimental conduct: MPB. Data analysis: MPB. Manuscript preparation: MPB, SPS. Both authors approved the final version of the manuscript.

## Conflict of Interest Statement

The authors declare that the research was conducted in the absence of any commercial or financial relationships that could be construed as a potential conflict of interest.
